# Improved Survival of Young Adults with Cancer Following the Passage of the Affordable Care Act

**DOI:** 10.1093/oncolo/oyab049

**Published:** 2022-02-01

**Authors:** Michael Roth, Amy Berkman, Clark R Andersen, Branko Cuglievan, J Andrew Livingston, Michelle Hildebrandt, Archie Bleyer

**Affiliations:** 1 Division of Pediatrics and Patient Care, The University of Texas MD Anderson Cancer Center, Houston, TX, USA; 2 Department of Pediatrics, Duke University School of Medicine, Durham, NC, USA; 3 Division of Biostatistics, The University of Texas MD Anderson Cancer Center, Houston, TX, USA; 4 Department of Sarcoma Medical Oncology, The University of Texas MD Anderson Cancer Center, Houston, TX, USA; 5 Department of Lymphoma and Myeloma, The University of Texas MD Anderson Cancer Center, Houston, TX, USA; 6 Department of Radiation Medicine, Oregon Health and Science University, Portland, OR, USA; 7 Department of Pediatrics, McGovern Medical School, University of Texas, TX, USA

**Keywords:** health insurance, cancer, young adult, Affordable Care Act

## Abstract

**Background:**

Compared with their ensured counterparts, uninsured adolescents and young adults (AYAs) with cancer are more likely to present with advanced disease and have poor prognoses. The Patient Protection and Affordable Care Act (ACA), enacted in 2010, provided health care coverage to millions of uninsured young adults by allowing them to remain on their parents’ insurance until age 26 years (the Dependent Care Expansion, DCE). The impact of the expansion of insurance coverage on survival outcomes for young adults with cancer has not been assessed.

**Participants:**

Utilizing the Surveillance, Epidemiology, and End Results database, we identified all patients aged 12-16 (younger-AYAs), 19-23 (middle-AYAs), and 26-30 (older-AYAs) who were diagnosed with cancer between 2006-2008 (pre-ACA) and 2011-2013 (post-ACA).

**Methods:**

In this population-based cohort study, we used an accelerated failure time model to assess changes in survival rates before and after the enactment of the ACA DCE.

**Results:**

Middle-AYAs ages 19-23 (thus eligible to remain on their parents’ insurance) experienced significantly increased 2-year survival after the enactment of the ACA DCE (survival time ratio 1.25, 95% confidence interval: 0.75-2.43, *P* = .029) and that did not occur in younger-AYAs (ages 12-16). Patients with sarcoma and acute myeloid leukemia accounted for the majority of improvement in survival. Middle-AYAs of hispanic ethnicity and those with low socioeconomic status experienced trends of improved survival after the ACA DCE was enacted.

**Conclusion:**

Survival outcomes improved for young adults with cancer following the expansion of health insurance coverage. Efforts are needed to expand coverage for the millions of young adults who do not have health insurance.

## Teaser

A decade has passed since the implementation of the Patient Protection and Affordable Care Act, which included a Dependent Coverage Expansion provision for young adults. This article reports evidence of the benefit of this expanded coverage on cancer mortality.

**Implications for Practice**

Survival outcomes for young adult patients living with cancer improved significantly after enactment of the Affordable Care Act Dependent Coverage expansion. There were notable improvements for vulnerable populations including minorities and socioeconomically disadvantaged. These findings demonstrate that expanded access to insurance coverage may lead to immediate improvements in survival for patients with cancer and support the need for continued expansion of health insurance coverage, particularly for at-risk populations.

## Introduction

Survival rates of adolescents and young adults (AYAs; aged 15-39 years) with cancer have improved over the past few decades; however, disparities persist in the care and outcomes of AYAs compared with childhood patients living with cancer.^1-3^ These disparities are likely due to multiple factors, including differences in cancer biology, tolerance of intensive chemotherapy, location of care, and clinical trial enrollment, as well as differences in insurance coverage and access to care.^4-10^ In the US, the health insurance rate of young adults is dramatically lower than that of individuals younger than 18 years, and young adults in the US have less insurance and access to health care than those in any other socioeconomically advantaged country.^11^ The lack of health insurance hinders many AYAs’ ability to access quality health care, ultimately leading to longer times to diagnosis, more advanced disease at diagnosis, and poor survival among uninsured AYAs compared with those with insurance.^12-17^

Signed into US law in 2010, the Patient Protection and Affordable Care Act (ACA) includes a Dependent Coverage Expansion (DCE) provision that expanded health care coverage for young adults by permitting dependents up to age 26 years to remain on their parents’ health insurance plans.^18^ Prior to this, young adults were removed from parental insurance after age 18 and more than one-third of all young adults in their early 20s were uninsured. During the first 15 months after the ACA DCE’s passage, 2.5 million individuals age 19-25 years gained health insurance coverage through their parents’ policies.^11,19^ Uninsured rates among young adults continued to drop, and today, <18% of those in their early 20s are uninsured.^20^ In addition, minority young adults, who have one of the highest uninsured rates, had greater increases in coverage than non-minority young adults did.^21^

Patients living with cancer have also benefitted from the ACA DCE. By spring 2016, the uninsured rate of patients with cancer age 19-25 years was significantly lower than it was prior to 2010; in addition, the increase in the proportion of these patients who were diagnosed with stage I disease was significantly higher than that of older patients, who were not affected by the ACA DCE.^22^ The impact of the ACA DCE on survival for young adults diagnosed with cancer has not been previously assessed. As it is now a decade since ACA DCE implementation, we sought to determine whether there is evidence of benefit of expanded health insurance coverage from AYA DCE on cancer mortality.

## Methods

We extracted survival data from the National Cancer Institute’s Surveillance, Epidemiology, and End Results (SEER) 18 database for all patients diagnosed with invasive cancer at ages 12-16, 19-23 and 26-30 years between the years 2006-2008 and 2011-2013 using SEER∗Stat software (version 8.3.6).^23^ A county-level socioeconomic deprivation index (SES index) was defined based upon county-level variables following Truong et al^24^ (Supplementary Methods) To focus on the impact of the ACA DCE on survival and ensure comparability among age groups and pre- and post-ACA DCE time periods, the age groups and study periods were defined as follows. Pre- and post-ACA DCE time periods were defined as the years 2006-2008 and 2011-2013, respectively. Three age groups were defined, each spanning 5 years of age of diagnosis, with 2 years of follow-up (those surviving beyond 2 years were censored at 2 years) for all patients were defined as:

**Table:** 

Age group	Age span (at diagnosis)	Maximum age at follow-up
Younger-AYA (younger control)	12-16	18
Middle-AYA	19-23	25
Older-AYA (older control)	26-30	32

The Middle-AYA group includes the range of ages plus follow-up which qualified for inclusion in their parents’ insurance plan following the ACA. The Younger-AYA group qualified for inclusion in their parents’ insurance plan both prior to and following the ACA. The Older-AYA group did not qualify for inclusion in their parents’ insurance plan neither prior to nor following the ACA. Those aged 16-19 and 23-26 at diagnosis were excluded from discrete comparison groups as qualification for ACA DCE coverage for these patients changed during the follow-up period. The outcome of 2-year survival was chosen as this endpoint has previously been used in studies assessing the impact of insurance status on cancer mortality as well as defined as the transition point between active cancer treatment and survivorship care for most AYAs.^25-28^

Demographical characteristics were summarized overall and by ACA DCE time point and age group using descriptive statistics. Overall survival (OS) was summarized using the Kaplan-Meier method by age group, sex, race, and SES tertile, with relation to time point. Differences between time points were assessed by the log-rank test.

Accelerated failure time (AFT) models were utilized to model survival over time following diagnosis (Supplementary Methods).^29^ Survival following diagnosis was modeled with relation to age group (younger-AYA, middle-AYA, and older-AYA) and time point (pre-ACA DCE vs post-ACA DCE), including an interaction between age group and time point, with adjustment for demographic covariates, race, sex, rurality, SES index, and (where available) lymphoma Ann Arbor stage. Differences in survival among age groups by time point and among time points by age group were assessed by contrasts, with Hommel-adjusted *P*-values.^30^ There were separate models by disease condition, as well as a single overall model which included disease condition as a covariate, and excluded Ann Arbor stage.

To compare survival pre- and post- ACA DCE by race, in separate overall models by age group, survival following disease diagnosis was modeled with relation to race and time point, including an interaction between race and time point, with adjustment for demographic covariates disease condition, sex, rurality, and SES index. Differences in survival among races by time point and among time points by race were assessed by contrasts, with Hommel-adjusted *P*-values. A similar analysis was performed to compare survival by SES tertile, adjusting by demographic covariates, disease condition, sex, rurality, and race. Kaplan-Meier survival analysis was used to summarize and illustrate OS pre- and post-ACA DCE according to age group at diagnosis, sex, race/ethnicity, and SES tertile.

Statistical analyses were performed using R statistical software (R Core Team, 2020, version 3.6.3).^31^ In all statistical tests, 2-sided α= 0.05. Survival modeling was performed using the “survival” package.^32,33^ Assessment of differences among discrete variable levels in the AFT model were estimated using the “emmeans” package^34^; this includes adjusted means weighted proportionally to covariate marginal frequencies.

## Results

### Patient Population

The characteristics of 11 838 patients included in the analysis are shown in Table 1. Forty-five percent of the population was female. Racial/ethnic make-up of the population included 57% non-Hispanic White patients, 24% Hispanic patients, 12% non-Hispanic Black patients, and 7% non-Hispanic Asian or Pacific Islander patients. Hodgkin lymphoma was the most common diagnosis, with 3533 cases including 1816 diagnoses pre-ACA DCE and 1717 diagnoses post-ACA DCE. Diagnoses by cancer type, stratified by time period (pre- and post-ACA DCE) and age group (younger-AYA, middle-AYA, and older-AYA) are shown in Table 1 and further detailed in Supplementary Tables S1A–F.

**Table 1.** Patient characteristics.

Characteristic	Overall total, no. (%), N = 11 838	Total pre-ACA DCE, *N* = 5867	Total post-ACA DCE, *N* = 5971
Sex			
Male	6569 (55.5)		
Female	5269 (44.5)		
Race/ethnicity			
Hispanic (all races)	2872 (24.3)		
Non-Hispanic Asian or Pacific Islander	866 (7.3)		
Non-Hispanic Black	1385 (11.7)		
Non-Hispanic White	6715 (56.7)		
Socioeconomic Status Index Tertile			
Tertile 1 (highest SES)	3926 (33.2)		
Tertile 2	3960 (33.5)		
Tertile 3	3952 (33.4)		
Age group			
Younger-AYA (12-16 years)		1603 (27.3)	1546 (25.9)
Middle-AYA (19-23 years)		2080 (35.5)	2113 (35.4)
Older-AYA (26-30 years)		2184 (37.2)	2312 (38.7)
*Cancer type*			
Hodgkin lymphoma			
Total		1816	1717
Younger-AYA		329 (18.1)	250 (14.6)
Middle-AYA		768 (42.3)	759 (44.2)
Older-AYA		719 (39.6)	708 (41.2)
CNS tumors			
Total		1199	1276
Younger-AYA		341 (28.4)	344 (27.0)
Middle-AYA		393 (32.8)	408 (32.0)
Older-AYA		465 (38.8)	524 (41.1)
Non-Hodgkin lymphoma			
Total		1104	1226
Younger-AYA		224 (20.3)	209 (17.0)
Middle-AYA		351 (31.8)	405 (33.0)
Older-AYA		529 (47.9)	612 (49.9)
Acute lymphoblastic leukemia			
Total		662	648
Younger-AYA		325 (49.1)	318 (49.1)
Middle-AYA		193 (29.2)	195 (30.1)
Older-AYA		144 (21.8)	135 (20.8)
Sarcomas			
Total		549	556
Younger-AYA		274 (49.9)	302 (54.3)
Middle-AYA		184 (33.5)	152 (27.3)
Older-AYA		91 (16.6)	102 (18.3)
Acute myeloid leukemia			
Total		537	548
Younger-AYA		110 (20.5)	123 (22.4)
Middle-AYA		191 (35.6)	194 (35.4)
Older-AYA		236 (43.9)	231 (42.2)

### Unadjusted Change in Survival Following ACA by Age, Sex, Race/Ethnicity, and SES

To specifically assess the survival benefit after introduction of the ACA DCE, we categorized AYAs into distinct diagnosis age groups, chosen to discretely stratify into ages that did or did not gain coverage through parental insurance with the ACA DCE. These included younger-AYAs (aged 12-16 years, eligible for parental insurance coverage pre- and post-ACA DCE), middle-AYAs (aged 19-23 years, eligible to gain parental insurance coverage through ACA DCE), and older-AYAs (aged 26-30 years, not eligible for parental insurance coverage pre- and post-ACA DCE). Adolescents and young adults that could have gained (age 16-19 at diagnosis) or lost (age 23-26 at diagnosis) ACA DCE coverage during the follow-up period were excluded.

**Figure 1.**
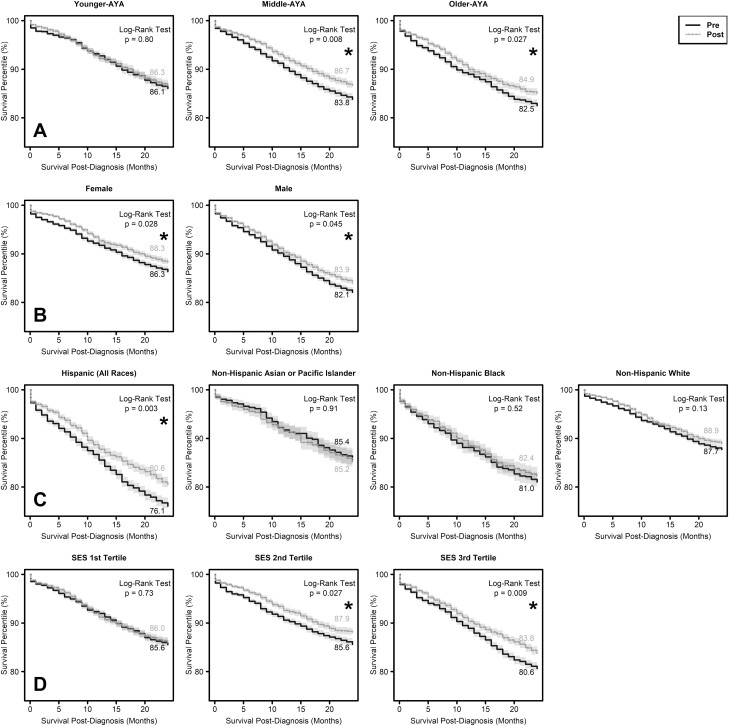
Unadjusted Kaplan-Meier summaries of overall survival comparing diagnoses pre- and post- enactment of the Affordable Care Act Dependent Coverage Expansion (ACA DCE). (**A**) Overall survival estimates by age group. The overall survival estimates of middle-AYAs and older-AYAs were higher for those diagnosed post-ACA DCE. There were no differences in survival estimates for younger-AYAs. (**B**) Overall survival estimate for all AYA age categories combined by sex. The overall survival estimates for both females and males diagnosed post-ACA DCE was higher than those diagnosed pre-ACA DCE. (**C**) Overall survival estimates for all AYA age categories combined by race/ethnicity. Overall survival estimate for Hispanics diagnosed post-ACA DCE was higher than that for Hispanics diagnosed pre-ACA DCE. There were no differences in overall survival estimates pre- and post-ACA DCE for non-Hispanic Asian or Pacific Islanders, non-Hispanic Blacks, or non-Hispanic Whites. (**D**) Overall survival estimates for all AYA age categories combined by socioeconomic status (SES) tertile. There were no differences in overall survival estimates pre- and post-ACA DCE for those in the highest (tertile 1) SES category. Patients in the middle (tertile 2) and lowest (tertile 3) SES categories had improved survival post-ACA DCE.

Survival by age group is shown in Fig. 1A. Survival of all AYA age categories combined by sex, race/ethnicity, and SES tertile is shown by diagnosis period pre- and post- ACA DCE through 24 months of follow-up in the Kaplan-Meier curves in Fig. 1B-D. There was no significant evidence of change in survival for younger-AYAs diagnosed pre- and post-ACA DCE (Fig. 1A, *P* = .80). Middle-AYAs diagnosed post-ACA DCE had significantly improved survival compared with those diagnosed pre-ACA DCE (*P* = .008). Older-AYAs diagnosed post-ACA DCE also had improved survival (*P* = .027). Survival improved for both females (*P* = .028) and males (*P* = .045) diagnosed post-ACA DCE compared with those diagnosed prior (Fig. 1B). Of the race/ethnicity groups assessed, Hispanic patients diagnosed post-ACA DCE had significantly improved survival compared with those diagnosed pre-ACA DCE (*P* = .003), while there were no survival differences for Non-Hispanic Asian or Pacific Islander, non-Hispanic Black, or non-Hispanic White patients (Fig. 1C). Those in the highest SES tertile did not have significant changes in survival pre- vs post-ACA DCE (*P* = .73), while there were significant improvements for those in the middle (*P* = .027) and lowest (*P* = .009) tertiles (Fig. 1D).

There were no pre- and post-ACE DCE survival differences for younger-AYAs when categorized by sex, race/ethnicity, or SES tertile (Supplementary Fig. S1). Female middle-AYAs had improved survival post-ACA DCE compared with pre-ACA DCE (*P* = .039), while males did not (Supplementary Fig. S2B). Of the race/ethnicity groups assessed, Hispanic middle-AYAs had improved survival post-ACA DCE (*P* = .001), while there were no significant pre- and post-ACA DCE survival changes for other race/ethnicity groups assessed (Supplementary Fig. S2C). There was improved survival post-ACA DCE for middle-AYAs in the lowest SES tertile (*P* = .023), but not for those in the highest or middle SES tertiles (Supplementary Fig. S2D). Similar to middle-AYAs, older-AYA females experienced improved survival post-ACE DCE compared with pre-ACE DCE (*P* = .09) while males had no significant survival changes (Supplementary Fig. S3B). White older-AYAs were the only race/ethnicity group in this age category to experience improvement in survival post- compared with pre-ACA DCE (*P* = .04, Supplementary Fig. S3C). Also similar to middle-AYAs, older AYAs in the lowest SES tertile were the only SES group to experience improved survival post-ACA DCE (*P* = .024, Supplementary Fig. S3D).

### Change in Survival Following ACA by Age Group on Multivariate Adjusted Analysis

Multivariate analyses made adjustments for demographic covariates, disease condition, sex, rurality, SES index, and race. Overall, younger-AYAs had no significant change in 2-year survival post-ACA DCE compared with pre-ACA DCE (Table 2, survival time ratio (STR: 1.12, 95% confidence interval (CI): 0.87-1.44). Those diagnosed as middle-AYAs had the greatest survival benefit associated with the introduction of the ACA DCE, with a 35% increased survival time (STR: 1.35, 95% CI: 1.09-1.67, Hommel *P* = .029) for those diagnosed after ACA DCE passage compared with those diagnosed prior. Older-AYAs diagnosed post-ACA DCE showed a non-significant trend of survival advantage compared with those diagnosed prior (STR: 1.27, 95% CI: 1.05-1.55, Hommel *P* = .062).

**Table 2.** Change in 2-year survival pre- and post-ACA DCE by AYA age category and cancer type.

AYA age cancer type and age category	Survival time ratio (95% confidence interval)	Unadjusted *P*-value	Hommel *P*-value^a^
Overall			
Younger-AYA (12-16 years)	1.12 (0.87-1.44)	.380	.380
Middle-AYA (19-23 years)	1.35 (1.09-1.67)	**.006**	**.029**
Older-AYA (26-30 years)	1.27 (1.05-1.55)	**.015**	.062
Hodgkin lymphoma			
Younger-AYA	2.57 (0.15-43.08)	.510	.710
Middle-AYA	1.16 (0.52-2.61)	.710	.710
Older-AYA	0.73 (0.32-1.64)	.440	.710
CNS tumors			
Younger-AYA	1.18 (0.77-1.81)	.440	.950
Middle-AYA	0.89 (0.59-1.34)	.580	.950
Older-AYA	1.10 (0.77-1.57)	.600	.950
Non-Hodgkin lymphoma			
Younger-AYA	2.62 (0.93-7.41)	.069	.390
Middle-AYA	1.44 (0.77-2.68)	.250	.500
Older-AYA	1.30 (0.80-2.81)	.290	.530
Acute lymphoblastic leukemia			
Younger-AYA	1.24 (0.72-2.13)	.430	.490
Middle-AYA	1.18 (0.73-1.90)	.490	.490
Older-AYA	1.33 (0.82-2.16)	.250	.490
Sarcomas			
Younger-AYA	0.97 (0.75-1.25)	.830	.870
Middle-AYA	1.53 (1.12-2.09)	**.008**	**.042**
Older-AYA	1.03 (0.73-1.44)	.870	.870
Acute myeloid leukemia			
Younger-AYA	0.75 (0.36-1.58)	.450	.850
Middle-AYA	1.84 (1.07-3.16)	**.028**	.170
Older-AYA	2.00 (1.20-3.32)	**.008**	.066

*P*-values <.05 are highlighted in bold.

Adjusted *P*-values to compensate for multiple comparisons among levels of discrete covariates.

### Change in Survival Following ACA by Age Group, Race/Ethnicity and SES on Multivariate Analysis

When assessing the change in survival within race/ethnicity groups by previously described discrete age categories in the multivariate analysis (Table 3), we found that Hispanic middle-AYA patients with cancer had the greatest trend in improvement in 2-year survival post-ACA DCE compared with pre-ACA DCE, although this lacked significance following adjustment for multiple comparisons (STR: 1.58, 95% CI: 1.13-2.22, Hommel *P* = .10). Younger-AYAs showed a similar trend in improvement (STR: 1.53, 95% CI: 1.04-2.25, Hommel *P* = .29). There were no significant differences in pre- and postsurvival by age category among non-Hispanic Black patients, nor among non-Hispanic Asian or Pacific Islanders. Among non-Hispanic White patients, older-AYAs showed a trend in improvement in 2-year survival post-ACA DCE compared with prior, although this lost significance following adjustment for multiple comparisons (STR: 1.38, 95% CI: 1.00-1.90, Hommel *P* = .49).

**Table 3.** Change in 2-year survival pre- and post-ACA DCE by race/ethnicity and AYA age category.

Race/ethnicity and AYA age category	Survival time ratio (95% confidence interval)	Unadjusted *P*-value	Hommel *P*-value^a^
Hispanic (all races)			
Younger-AYA	1.53 (1.04-2.25)	**.029**	.290
Middle-AYA	1.58 (1.13-2.22)	**.008**	.100
Older-AYA	1.14 (0.78-1.67)	.510	.970
Non-Hispanic Black			
Younger-AYA	1.14 (0.63-2.07)	.660	.970
Middle-AYA	1.17 (0.69-1.99)	.560	.970
Older-AYA	1.59 (0.90-2.81)	.110	.880
Non-Hispanic White			
Younger-AYA	0.91 (0.66-1.26)	.570	.970
Middle-AYA	1.21 (0.88-1.65)	.240	.970
Older-AYA	1.38 (1.00-1.90)	**.049**	.490
Non-Hispanic Asian or Pacific Islander			
Younger AYA	1.01 (0.44-2.34)	.970	.970
Middle-AYA	1.37 (0.63-3.02)	.430	.970
Older-AYA	0.95 (0.43-2.06)	.890	.970

*P*-values <.05 are highlighted in bold.

Adjusted *P*-values to compensate for multiple comparisons among levels of discrete covariates.

To determine the impact of the AYA DCE on 2-year survival by county-level SES and AYA age category, we divided SES into tertiles, with tertile 1 (T1) representing the highest SES level and tertile 3 (T3) the lowest. In the multivariate analysis, middle-AYAs in both T2 and T3 experienced trends towards improved 2-year survival after the introduction of the ACA DCE (STR: 1.49, 95% CI: 1.03-2.15, Hommel *P* = .23 and STR: 1.46, 95% CI: 1.05-2.02, Hommel *P* = .16, respectively), while middle-AYAs in T1 did not experience significant change in 2-year relative survival (Table 4).

**Table 4.** Change in 2-year survival pre- and post-ACA DCE by SES and AYA age category.

SES tertile and AYA age category	Survival time ratio (95% confidence interval)	Unadjusted *P*-value	Hommel *P*-value^a^
Tertile 1 (highest SES)			
Younger-AYA	1.02 (0.70-1.49)	.920	.920
Middle-AYA	1.06 (0.73-1.53)	.770	.920
Older-AYA	1.07 (0.73-1.57)	.730	.920
Tertile 2			
Younger-AYA	1.35 (0.90-2.02)	.140	.720
Middle-AYA	1.49 (1.03-2.15)	**.033**	.230
Older-AYA	1.35 (0.89-2.04)	.160	.790
Tertile 3 (lowest SES)			
Younger-AYA	1.06 (0.73-1.53)	.760	.920
Middle-AYA	1.46 (1.05-2.02)	**.023**	.160
Older-AYA	1.46 (1.04-2.05)	**.029**	.200

*P*-values <.05 are highlighted in bold.

Adjusted *P*-values to compensate for multiple comparisons among levels of discrete covariates.

### Change in AYAs’ Survival Rates by Type of Invasive Cancer

On multivariate analysis of discrete age categories, diagnoses of AML (STR: 1.53, 95% CI: 1.12-2.09, Hommel *P* = .042) and sarcoma (STR: 1.84, 95% CI: 1.07-3.26, Hommel *P* = .17, Table 2) drove the survival benefit of the ACA DCE for middle-AYAs. Middle-AYAs with sarcoma and AML diagnosed post-ACA DCE had ~50% and ~80% survival time increases, respectively, compared with those diagnosed pre-ACA DCE.

## Discussion

Our findings show that following the ACA DCE, a policy aimed at increasing insurance coverage among young adults, there were improved early relative survival outcomes after an invasive cancer diagnosis in AYAs. Importantly, the age subset within they population most impacted by the law had the greatest increase in survival following enactment, which suggests that increased access to health insurance coverage has had a meaningful impact on the long-term survival of AYAs with cancer.

Compared with those who have health insurance, AYAs who do not have health insurance are more likely to experience delays in diagnosis and present with locally advanced or metastatic disease.^13,16,17^ For many patients with cancer, the presence of metastatic disease significantly worsens prognosis; thus, delays in diagnosis and treatment directly impact cure rates, and data has shown that after the implementation of the ACA DCE, eligible AYAs with colon cancer presented with earlier stages of disease, compared to prior to ACA DCE implementation.^35,36^ The ACA DCE has also been shown to decrease insurance interruptions for AYA patients with cancer.^37^ Shorter times to diagnosis and continuity of insurance and thus care likely contributed to the improved survival rates of DCE-eligible AYAs.

When assessing survival by cancer type, middle-AYAs with sarcoma and AML derived the greatest survival increases post-ACA DCE compared with pre-ACA DCE. These 2 AYA cancer diagnoses are particularly aggressive, with even short delays in treatment resulting in significantly reduced survival compared with patients with no treatment delay.^38-40^ Longer follow-up time might reveal additional survival benefits for patients with malignancies that are characterized by later relapses. Additionally, delays in the diagnosis of some cancers such as Hodgkin lymphoma can result in patients presenting with advanced disease necessitating more aggressive treatments, such as radiation therapy,^11^ that are associated with a high risk of treatment-related late effects and late mortality.^41-45^ Earlier stage at diagnosis and resultant diminished toxicity can be expected to not only improve quality of life but also reduce the financial burden of healthcare. Lack of insurance alone is associated with increased risk of treatment related late effects among AYA cancer survivors,^46,47^ and it will be important to determine the impact that implementation of the ACA DCE has on long-term late effects and quality of life among AYA cancer survivors.

Black and Hispanic individuals are more than twice as likely as White individuals to be uninsured.^48^ The ACA has reduced ethnic and racial disparities in health insurance coverage for black and Hispanic populations.^48^ This same pattern holds true in the AYA cancer population, with a larger increase in insurance coverage among Hispanic AYAs compared with other racial/ethnic groups.^49-51^ Minority AYA patients with cancer have higher cancer-specific mortality rates than non-minority patients with cancer do; however, the impact of expanding insurance coverage on this survival disparity has not been assessed previously.^52^ We found that both Hispanic and non-Hispanic black AYAs who were eligible for the ACA DCE had significantly greater improvements in short-term relative survival than non-Hispanic White AYAs did, which suggests that expanding access to insurance coverage can aid in reducing race/ethnicity survival disparities. Importantly, increasing access to insurance coverage appears to rapidly narrow the gap in survival outcomes between minority and non-minority AYA patients with cancer, similarly to previous data in the childhood cancer population showing that health insurance attenuates racial/ethnic disparities in cancer mortality.^53^

Compared with higher SES status, AYA patients with cancer with low SES status have increased risk of mortality, ^3,54,55^ with health insurance attenuating this risk.^56^ We found that enactment of the AYA DCE was associated with significantly improved 2-year survival among middle-AYAs in the bottom 2 tertiles of SES, while survival was not impacted for those in the highest SES tertile, showing that improved access to insurance coverage can narrow SES disparities in AYA cancer survival.

One limitation of this study is that the insurance statuses of individuals included are unknown, including whether the patients in this cohort specifically gained coverage through the ACA DCE is unknown. Additionally, it is unknown whether the individual AYA patients with cancer who gained health insurance through the DCE accounted for the significant increases in survival after the implementation of the ACA. Detailed treatment information is unknown, and while it is unlikely that over the short time frame from pre- to post-ACA DCE new treatments were developed that differentially impacted subgroups of ages within the AYA population, it is possible. Finally, the population assessed was based on a sample of the US (18 regions covered by SEER) instead of the entire US, albeit the regions cover approximately 25% of the country’s population and were selected to be racially, ethnically, socioeconomically, and geographically representative of the country.

In conclusion, our findings demonstrate that survival outcomes of AYA patients with cancer significantly improved shortly after the implementation of the ACA DCE in 2010. They also demonstrate that expanded access to insurance coverage can result in immediate, impactful improvements in outcomes and support broader implementation of the ACA including further expansion of the DCE provision. Additional provisions of the ACA, such as the newly passed measure promoting equitable access to clinical trial participation,^57^ also hold promise to improve survival in AYA patients with cancer.

## Supplementary Material

oyab049_suppl_Supplementary_Figures

oyab049_suppl_Supplementary_Tables
