# Supplementary material for: Improved Survival of Young Adults with Cancer Following the Passage of the Affordable Care Act
Source: Oncologist. 2022 Feb 1;27(2):135–43. doi: 10.1093/oncolo/oyab049 (PMC8895735; doi:10.1093/oncolo/oyab049)
Supplement: oyab049_suppl_Supplementary_Figures [file oyab049_suppl_supplementary_figures.pdf]

The Oncologist
The official journal of the Society for Translational Oncology

Supplemental Figures for:
Improved Survival of Young Adults with Cancer Following the Passage of the Affordable Care Act
Michael Roth et al.

Supplemental Figures:

Supplemental Figure 1. Unadjusted Kaplan-Meier summaries of overall survival comparing diagnoses pre- and post- enactment of the Affordable Care Act Dependent Coverage Expansion (ACA DCE) for younger-AYAs (age 12-16 at diagnosis). **A)** Overall survival estimates. There was no difference in survival estimate for younger-AYAs diagnosed pre- and post-ACA DCE. **B)** Overall survival estimates for younger-AYAs by sex. The overall survival estimates for both females and males diagnosed post-ACA DCE were similar to those diagnosed pre-ACA DCE. **C)** Overall survival estimates for younger-AYAs by race/ethnicity. There were no differences in overall survival estimates pre- and post-ACA DCE for Hispanic, non-Hispanic Asian or Pacific Islander, non-Hispanic Black, or non-Hispanic White younger-AYAs. **D)** Overall survival estimates for all younger-AYAs socioeconomic status (SES) tertile. There were no differences in overall survival estimates pre- and post-ACA DCE in any SES tertile.

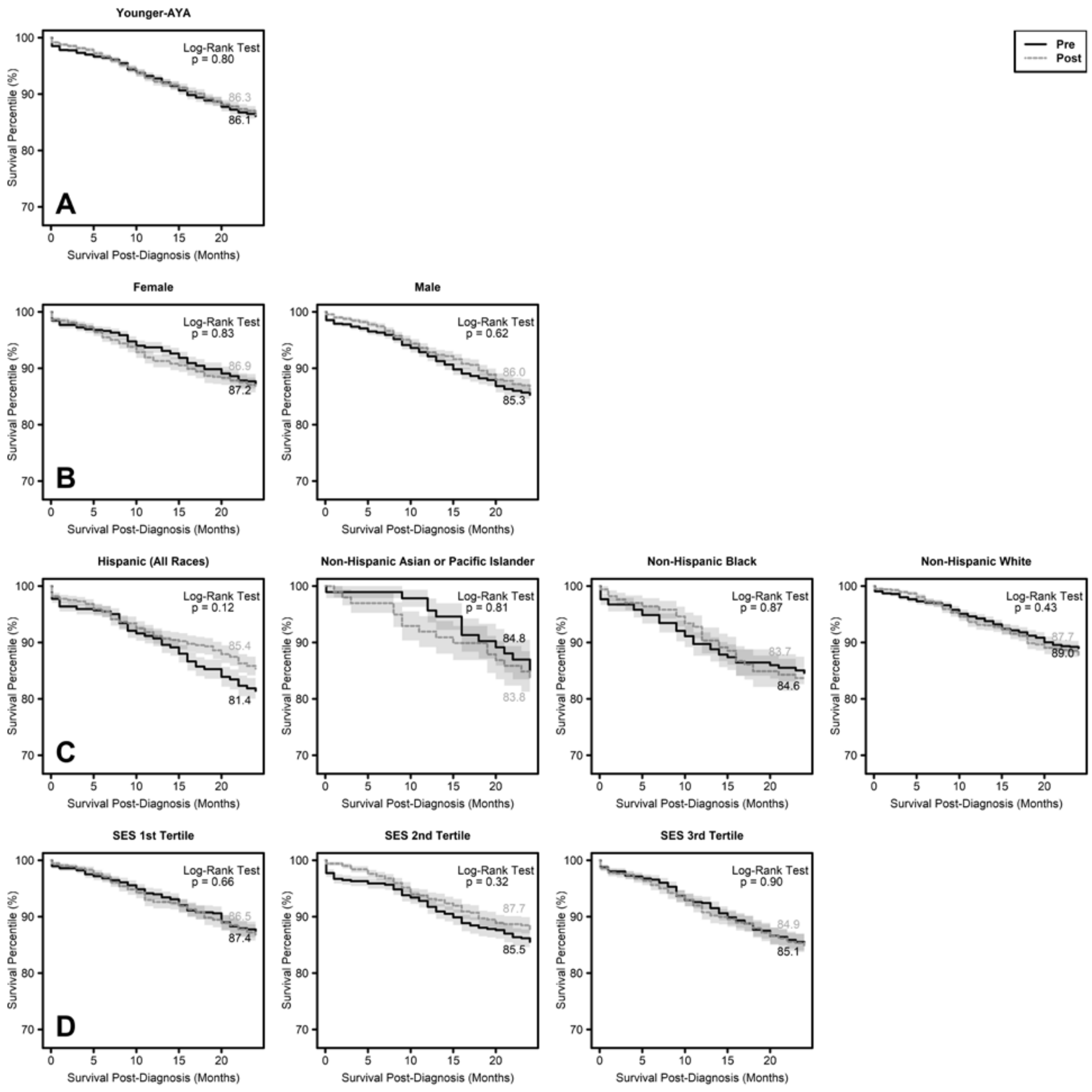

Younger-AYA
Log-Rank Test
p = 0.80
86.3
86.1
A
Female
Log-Rank Test
p = 0.83
86.9
87.2
B
Male
Log-Rank Test
p = 0.62
86.0
85.3
Hispanic (All Races)
Log-Rank Test
p = 0.12
85.4
81.4
C
Non-Hispanic Asian or Pacific Islander
Log-Rank Test
p = 0.81
84.8
83.8
Non-Hispanic Black
Log-Rank Test
p = 0.87
83.7
84.6
Non-Hispanic White
Log-Rank Test
p = 0.43
87.7
89.0
SES 1st Tertile
Log-Rank Test
p = 0.66
86.5
87.4
D
SES 2nd Tertile
Log-Rank Test
p = 0.32
87.7
85.5
SES 3rd Tertile
Log-Rank Test
p = 0.90
84.9
85.1
Pre
Post
Survival Percentile (%)
Survival Post-Diagnosis (Months)

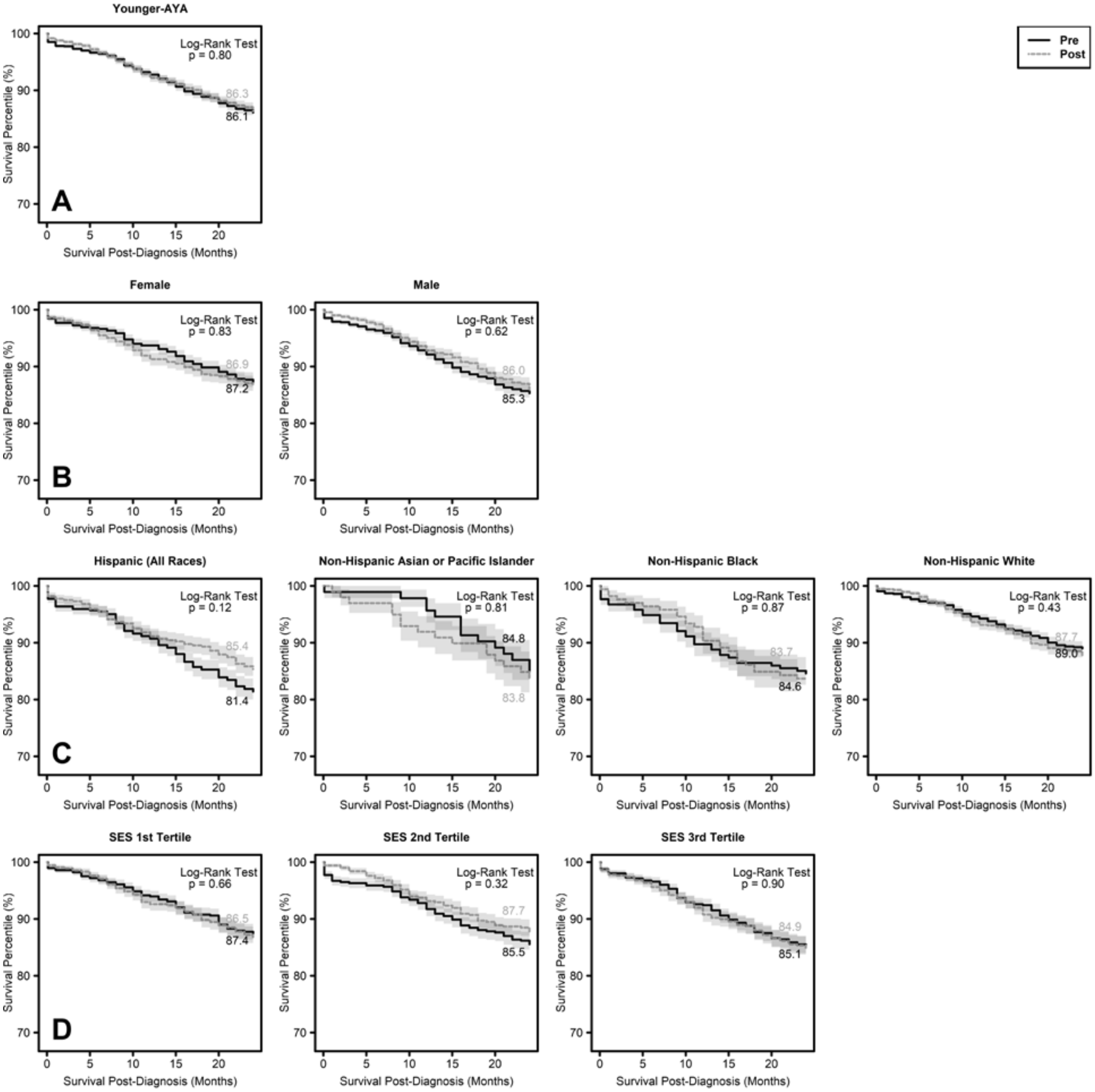

Younger-AYA
Log-Rank Test p = 0.80
86.3
86.1
A
Female
Log-Rank Test p = 0.83
86.9
87.2
B
Male
Log-Rank Test p = 0.62
86.0
85.3
Hispanic (All Races)
Log-Rank Test p = 0.12
85.4
81.4
C
Non-Hispanic Asian or Pacific Islander
Log-Rank Test p = 0.81
84.8
83.8
Non-Hispanic Black
Log-Rank Test p = 0.87
83.7
84.6
Non-Hispanic White
Log-Rank Test p = 0.43
87.7
89.0
SES 1st Tertile
Log-Rank Test p = 0.66
86.5
87.4
D
SES 2nd Tertile
Log-Rank Test p = 0.32
87.7
85.5
SES 3rd Tertile
Log-Rank Test p = 0.90
84.9
85.1
Survival Percentile (%)
Survival Post-Diagnosis (Months)
Pre
Post

Supplemental Figure 2. Unadjusted Kaplan-Meier summaries of overall survival comparing diagnoses pre- and post- enactment of the Affordable Care Act Dependent Coverage Expansion (ACA DCE) for middle-AYAs (age 19-23 at diagnosis). **A)** Overall survival estimates. The overall survival estimate for middle-AYAs diagnosed post-ACA DCE was higher survival compared with those diagnosed pre-ACA DCE. **B)** Overall survival estimates for middle-AYAs by sex. The overall survival estimate for females diagnosed post-ACA DCE was higher and the overall survival estimate for males diagnosed post-ACA DCE was similar to those diagnosed pre-ACA DCE. **C)** Overall survival estimates for middle-AYAs by race/ethnicity. Overall survival estimate for Hispanics diagnosed post-ACA DCE was higher than that for Hispanics diagnosed pre-ACA DCE. There were no differences in overall survival estimates pre- and post-ACA DCE for non-Hispanic Asian or Pacific Islanders, non-Hispanic Blacks, or non-Hispanic Whites. **D)** Overall survival estimates for middle-AYAs by socioeconomic status (SES) tertile. There were no differences in overall survival estimates pre- and post-ACA DCE for those in the highest (tertile 1) and the middle (tertile 2) SES tertiles. Middle-AYAs in the lowest (tertile 3) SES category had improved survival post-ACA DCE.

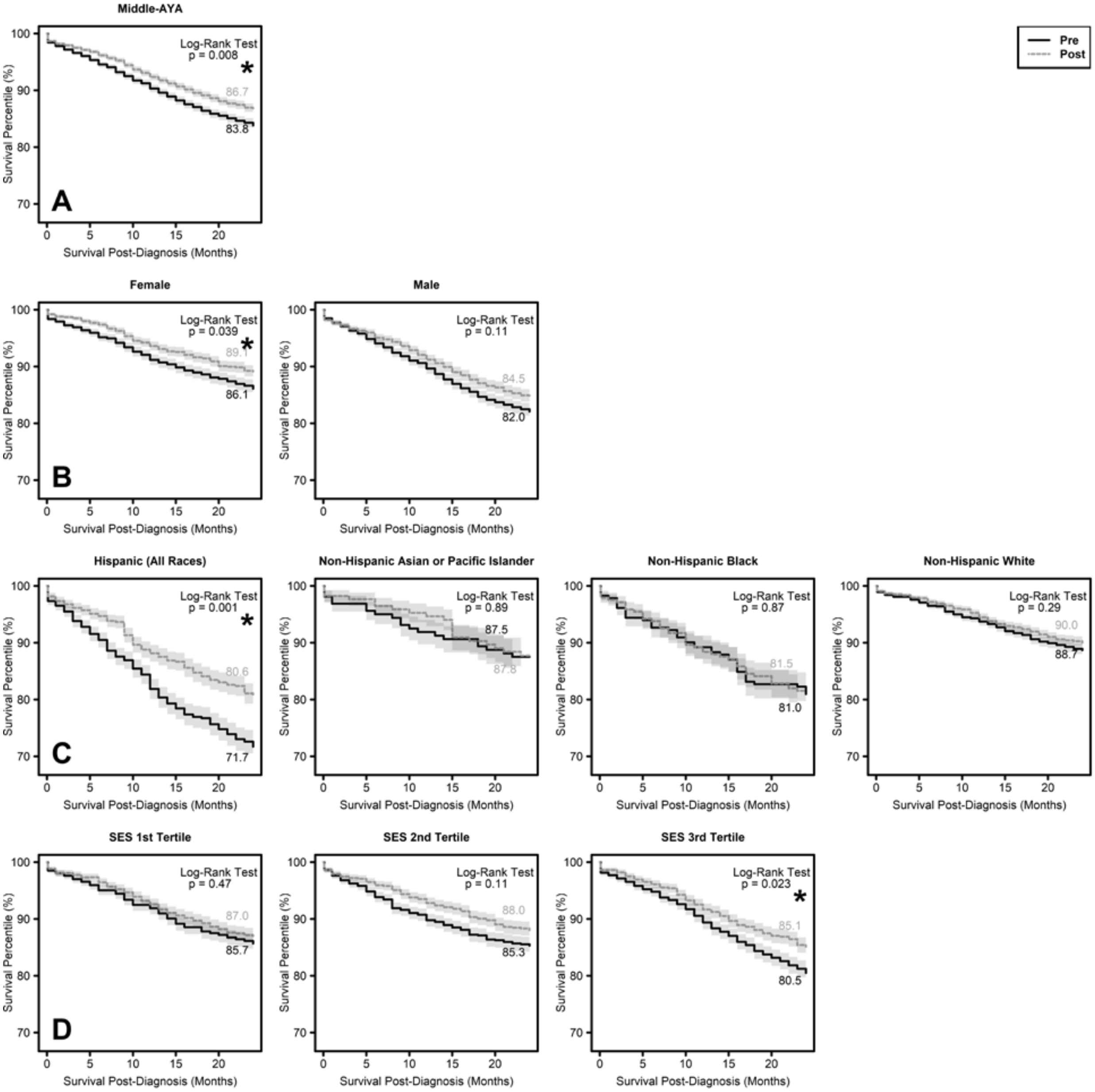

Middle-AYA
Log-Rank Test p = 0.008 *
86.7
83.8
A
Pre
Post
Female
Log-Rank Test p = 0.039 *
89.1
86.1
B
Male
Log-Rank Test p = 0.11
84.5
82.0
Hispanic (All Races)
Log-Rank Test p = 0.001 *
80.6
71.7
C
Non-Hispanic Asian or Pacific Islander
Log-Rank Test p = 0.89
87.5
87.8
Non-Hispanic Black
Log-Rank Test p = 0.87
81.5
81.0
Non-Hispanic White
Log-Rank Test p = 0.29
90.0
88.7
SES 1st Tertile
Log-Rank Test p = 0.47
87.0
85.7
D
SES 2nd Tertile
Log-Rank Test p = 0.11
88.0
85.3
SES 3rd Tertile
Log-Rank Test p = 0.023 *
85.1
80.5
Survival Percentile (%)
Survival Post-Diagnosis (Months)

Supplemental Figure 3. Unadjusted Kaplan-Meier summaries of overall survival comparing diagnoses pre- and post- enactment of the Affordable Care Act Dependent Coverage Expansion (ACA DCE) for older-AYAs (age 26-30 at diagnosis). **A)** Overall survival estimates. The overall survival estimate for older-AYAs diagnosed post-ACA DCE was higher survival compared with those diagnosed pre-ACA DCE. **B)** Overall survival estimates for older-AYAs by sex. The overall survival estimate for females diagnosed post-ACA DCE was higher and the overall survival estimate for males diagnosed post-ACA DCE was similar to those diagnosed pre-ACA DCE. **C)** Overall survival estimates for older-AYAs by race/ethnicity. Overall survival estimate for non-Hispanic Whites diagnosed post-ACA DCE was higher than that for non-Hispanic Whites diagnosed pre-ACA DCE. There were no differences in overall survival estimates pre- and post-ACA DCE for Hispanics, non-Hispanic Asian or Pacific Islanders, or non-Hispanic Blacks. **D)** Overall survival estimates for older-AYAs by socioeconomic status (SES) tertile. There were no differences in overall survival estimates pre- and post-ACA DCE for those in the highest (tertile 1) and the middle (tertile 2) SES tertiles. Older-AYAs in the lowest (tertile 3) SES category had improved survival post-ACA DCE.

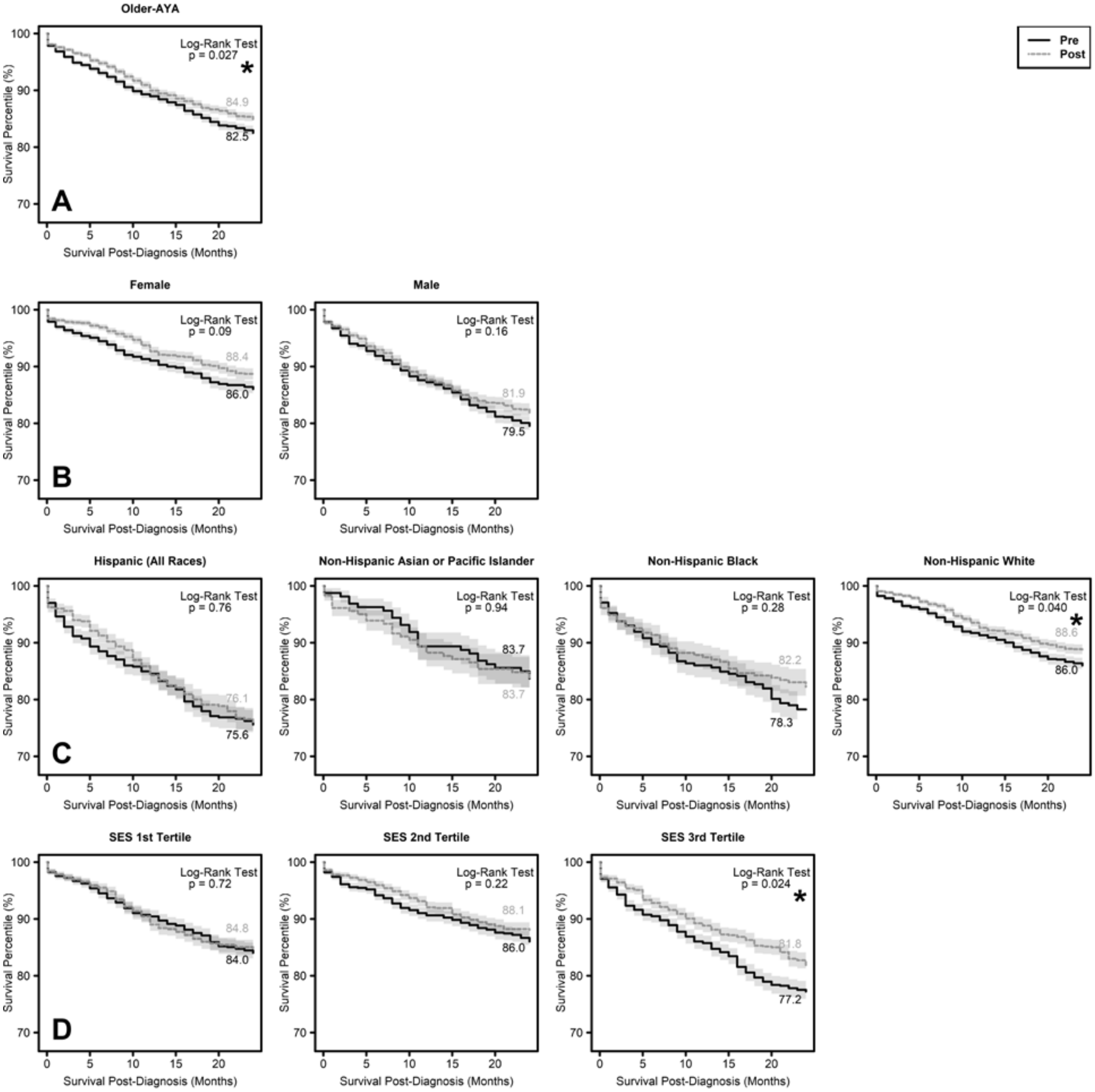

Older-AYA
Log-Rank Test p = 0.027 *
84.9
82.5
A
Pre
Post
Female
Log-Rank Test p = 0.09
88.4
86.0
B
Male
Log-Rank Test p = 0.16
81.9
79.5
Hispanic (All Races)
Log-Rank Test p = 0.76
76.1
75.6
C
Non-Hispanic Asian or Pacific Islander
Log-Rank Test p = 0.94
83.7
83.7
Non-Hispanic Black
Log-Rank Test p = 0.28
82.2
78.3
Non-Hispanic White
Log-Rank Test p = 0.040 *
88.6
86.0
SES 1st Tertile
Log-Rank Test p = 0.72
84.8
84.0
D
SES 2nd Tertile
Log-Rank Test p = 0.22
88.1
86.0
SES 3rd Tertile
Log-Rank Test p = 0.024 *
81.8
77.2
Survival Percentile (%)
Survival Post-Diagnosis (Months)
