# Supplementary material for: Improved Survival of Young Adults with Cancer Following the Passage of the Affordable Care Act
Source: Oncologist. 2022 Feb 1;27(2):135–43. doi: 10.1093/oncolo/oyab049 (PMC8895735; doi:10.1093/oncolo/oyab049)
Supplement: oyab049_suppl_Supplementary_Tables [file oyab049_suppl_supplementary_tables.pdf]

The Oncologist
The official journal of the Society for Translational Oncology

Supplemental Tables for:
Improved Survival of Young Adults with Cancer Following the Passage of the Affordable Care Act
Michael Roth et al.

Supplemental Methods

SES Index

A county-level socioeconomic deprivation index (SES index) was defined based upon county-level variables following Truong et al 2015. County-level variables used in defining the SES index included the level of poverty (P) based upon percentage with income below the 200th percentile of the poverty line, low educational attainment (E) per the percentage obtaining less than a high school education, crowding (C) per the percentage with room crowding with greater than one person per room, unemployment (U) per the percentage unemployed, levels of immigration (I) per the percentage of foreign-born, and language isolation (L) per the county percentage of language isolation. The variables P, E, C, U, I, L, were each standardized as the difference from the mean divided by the standard deviation. The county SES index was then calculated as (((P + E + C + U)/4) + ((I + L)/2))/2. Note that since this is defined as a socioeconomic *deprivation* index, higher values are indicative of greater deprivation. Additionally, to allow comparisons among tertiles of the SES index, tertiles T1, T2, and T3 were defined per the 1/3 and 2/3 quantiles of SES index as T1 where SES index < (-.49), T2 where SES index >= (-.4903995) and < (.2842182), and T3 where SES index >=(.2842182). Tertile 1 (T1) represents the highest SES level and tertile 3 (T3) the lowest.

Model Selection

Cox proportional hazards models had been considered, but were problematic due to extensive violations of the proportionality of hazards assumption. An advantage of utilizing accelerated failure time models is that survival time ratios provide an intuitive interpretation, in contrast to typical hazard ratios. The Weibull distribution was chosen as optimal for these survival models

based upon lower Akaike Information Criterion (AIC) and good fit of a Kaplan-Meier plot of model residuals to the assumed model distribution.

Supplemental Tables:

Supplemental Table 1A. Counts by Age Group and Diagnosis Period, Hodgkin Lymphoma

Characteristic	Younger-AYA, N (%)	Middle-AYA, N (%)	Older-AYA, N (%)	Chi-Square Test p-value
**Sex**				0.15
Pre-ACA DCE				
Female	153 (9)	377 (21)	371 (21)	
Male	176 (10)	391 (22)	348 (20)	
Post-ACE DCE				
Female	112 (6)	401 (23)	346 (20)	
Male	138 (8)	358 (20)	362 (20)	
**Race/Ethnicity**				0.059
Pre-ACE DCE				
Hispanic	77 (12)	124 (19)	126 (20)	
Non-Hispanic Asian or Pacific Islander	19 (8)	66 (27)	46 (19)	
Non-Hispanic Black	53 (12)	88 (20)	104 (24)	
Non-Hispanic White	180 (8)	490 (22)	443 (20)	
Post-ACA DCE				
Hispanic	52 (8)	157 (24)	107 (17)	
Non-Hispanic Asian or Pacific Islander	13 (5)	56 (23)	47 (19)	
Non-Hispanic Black	26 (6)	81 (18)	89 (20)	
Non-Hispanic White	159 (7)	465 (21)	465 (21)	
**Endpoint**				0.11
Pre-ACA DCE				
Censored	326 (9)	748 (22)	703 (20)	
Died	3 (4)	20 (26)	16 (21)	
Post-ACA DCE				
Censored	249 (7)	742 (21)	687 (20)	
Died	1 (1)	17 (22)	21 (27)	

Supplemental Table 1B. Counts by Age Group and Diagnosis Period, CNS Tumors

Characteristic	Younger-AYA, N (%)	Middle-AYA, N (%)	Older-AYA, N (%)	Chi-Square Test p-value
**Sex**				0.55
Pre-ACA DCE				
Female	153 (13)	179 (15)	207 (18)	
Male	188 (14)	214 (16)	258 (20)	
Post-ACE DCE				
Female	160 (14)	206 (18)	250 (22)	
Male	184 (14)	202 (15)	274 (21)	
**Race/Ethnicity**				0.68
Pre-ACE DCE				
Hispanic	73 (15)	80 (16)	94 (19)	
Non-Hispanic Asian or Pacific Islander	20 (14)	24 (16)	28 (19)	
Non-Hispanic Black	42 (19)	35 (16)	42 (19)	
Non-Hispanic White	206 (13)	254 (16)	301 (19)	
Post-ACA DCE				
Hispanic	69 (14)	74 (15)	100 (20)	
Non-Hispanic Asian or Pacific Islander	14 (10)	28 (19)	33 (22)	
Non-Hispanic Black	35 (16)	36 (16)	33 (15)	
Non-Hispanic White	226 (14)	270 (17)	358 (22)	0.78
**Endpoint**				
Pre-ACA DCE				
Censored	284 (13)	339 (16)	390 (19)	
Died	57 (16)	54 (15)	75 (20)	
Post-ACA DCE				
Censored	296 (14)	348 (17)	451 (21)	
Died	48 (13)	60 (16)	73 (20)	

Supplemental Table 1C. Counts by Age Group and Diagnosis Period, Non-Hodgkin Lymphoma

Characteristic	Younger-AYA, N (%)	Middle-AYA, N (%)	Older-AYA, N (%)	Chi-Square Test p-value
**Sex**				0.024
Pre-ACA DCE				
Female	73 (8)	119 (14)	204 (23)	
Male	151 (10)	232 (16)	325 (22)	
Post-ACE DCE				
Female	65 (7)	166 (19)	250 (29)	
Male	144 (10)	239 (16)	362 (25)	
**Race/Ethnicity**				0.002
Pre-ACE DCE				
Hispanic	52 (10)	79 (15)	121 (23)	
Non-Hispanic Asian or Pacific Islander	7 (3)	36 (17)	41 (20)	
Non-Hispanic Black	37 (10)	51 (14)	86 (24)	
Non-Hispanic White	128 (10)	185 (15)	281 (23)	
Post-ACA DCE				
Hispanic	63 (12)	76 (15)	128 (25)	
Non-Hispanic Asian or Pacific Islander	16 (8)	43 (21)	65 (31)	
Non-Hispanic Black	34 (9)	69 (19)	84 (23)	
Non-Hispanic White	96 (8)	217 (17)	335 (27)	
**Endpoint**				0.011
Pre-ACA DCE				
Censored	196 (10)	292 (15)	435 (22)	
Died	28 (8)	59 (17)	94 (27)	
Post-ACA DCE				
Censored	193 (10)	351 (18)	518 (26)	
Died	16 (5)	54 (16)	94 (27)	

Supplemental Table 1D. Counts by Age Group and Diagnosis Period, Acute Lymphoblastic Leukemia

Characteristic	Younger-AYA, N (%)	Middle-AYA, N (%)	Older-AYA, N (%)	Chi-Square Test p-value
**Sex**				0.060
Pre-ACA DCE				
Female	127 (26)	66 (13)	61 (12)	
Male	198 (24)	127 (16)	83 (10)	
Post-ACE DCE				
Female	135 (27)	58 (12)	50 (10)	
Male	183 (23)	137 (17)	85 (10)	
**Race/Ethnicity**				0.90
Pre-ACE DCE				
Hispanic	137 (23)	104 (17)	68 (11)	
Non-Hispanic Asian or Pacific Islander	17 (20)	13 (16)	8 (10)	
Non-Hispanic Black	32 (35)	12 (13)	7 (8)	
Non-Hispanic White	139 (26)	64 (12)	61 (11)	
Post-ACA DCE				
Hispanic	133 (22)	97 (16)	65 (11)	
Non-Hispanic Asian or Pacific Islander	23 (28)	14 (17)	8 (10)	
Non-Hispanic Black	23 (25)	8 (9)	9 (10)	
Non-Hispanic White	139 (26)	76 (14)	53 (10)	
**Endpoint**				0.0005
Pre-ACA DCE				
Censored	277 (28)	134 (13)	83 (8)	
Died	48 (16)	59 (19)	61 (20)	
Post-ACA DCE				
Censored	279 (28)	143 (14)	88 (9)	
Died	39 (13)	52 (17)	47 (15)	

Supplemental Table 1E. Counts by Age Group and Diagnosis Period, Sarcomas

Characteristic	Younger-AYA, N (%)	Middle-AYA, N (%)	Older-AYA, N (%)	Chi-Square Test p-value
**Sex**				0.22
Pre-ACA DCE				
Female	97 (22)	69 (16)	42 (10)	
Male	177 (26)	115 (17)	49 (7)	
Post-ACE DCE				
Female	117 (27)	61 (14)	49 (11)	
Male	185 (28)	91 (14)	53 (8)	
**Race/Ethnicity**				0.97
Pre-ACE DCE				
Hispanic	75 (25)	47 (16)	27 (9)	
Non-Hispanic Asian or Pacific Islander	20 (27)	12 (16)	8 (11)	
Non-Hispanic Black	32 (24)	28 (21)	11 (8)	
Non-Hispanic White	147 (25)	97 (16)	45 (8)	
Post-ACA DCE				
Hispanic	86 (28)	38 (13)	29 (10)	
Non-Hispanic Asian or Pacific Islander	20 (27)	9 (12)	5 (7)	
Non-Hispanic Black	35 (26)	18 (13)	11 (8)	
Non-Hispanic White	161 (27)	87 (15)	57 (10)	
**Endpoint**				0.0005
Pre-ACA DCE				
Censored	221 (26)	126 (15)	59 (7)	
Died	53 (20)	58 (22)	32 (12)	
Post-ACA DCE				
Censored	242 (29)	124 (15)	70 (8)	
Died	60 (23)	28 (11)	32 (12)	

Supplemental Table 1F. Counts by Age Group and Diagnosis Period, Acute Myeloid Leukemia

Characteristic	Younger-AYA, N (%)	Middle-AYA, N (%)	Older-AYA, N (%)	Chi-Square Test p-value
**Sex**				0.41
Pre-ACA DCE				
Female	48 (9)	102 (19)	111 (20)	
Male	62 (11)	89 (16)	125 (23)	
Post-ACE DCE				
Female	59 (11)	102 (19)	123 (23)	
Male	64 (12)	92 (17)	108 (20)	
**Race/Ethnicity**				0.25
Pre-ACE DCE				
Hispanic	28 (9)	55 (18)	67 (21)	
Non-Hispanic Asian or Pacific Islander	10 (9)	9 (8)	30 (28)	
Non-Hispanic Black	18 (13)	18 (13)	23 (17)	
Non-Hispanic White	54 (10)	109 (21)	116 (22)	
Post-ACA DCE				
Hispanic	40 (13)	51 (16)	73 (23)	
Non-Hispanic Asian or Pacific Islander	14 (13)	21 (20)	23 (21)	
Non-Hispanic Black	16 (12)	30 (22)	29 (22)	
Non-Hispanic White	53 (10)	92 (17)	106 (20)	
**Endpoint**				0.016
Pre-ACA DCE				
Censored	76 (11)	108 (16)	137 (20)	
Died	34 (9)	83 (21)	99 (25)	
Post-ACA DCE				
Censored	78 (11)	130 (19)	160 (23)	
Died	45 (11)	64 (16)	71 (18)	
